# IN TIME: WHAT IS THE STATUS OF THE CARE GIVEN TO CHILDREN WITH CANCER?

**DOI:** 10.1590/1984-0462/;2018;36;3;00019

**Published:** 2018

**Authors:** Antonio Sérgio Petrilli, Flavio Augusto Vercillo Luisi

**Affiliations:** a Department of Pediatrics, Escola Paulista de Medicina, Universidade Federal de São Paulo, São Paulo, SP, Brazil.; b Sector of Oncology/Discipline of Pediatric Specialties, Escola Paulista de Medicina, Universidade Federal de São Paulo, São Paulo, SP, Brazil.

In developed countries, when a child with cancer is cared for early, and receives adequate, accessible and complete treatment, the chances of cure are around 80%. However, most of the pediatric population is concentrated in underdeveloped or developing countries, and, in these locations, the chances of cure are less than 20%.^1^

In Brazil, we have made much progress in this area: in the past few years, the definition of childhood cancer as a public health issue enabled the contribution of the health services and the participation of the civil society in search for improving the understanding and the inclusion of childhood cancer in the means of communication, which was translated into the effort to build a care network addressed to oncology in children and adolescents, aiming at necessary actions; for instance, there are more than 20 ­population-based cancer registry centers, and these data help the public policies.^2^

Besides, the Brazilian Society of Pediatric Oncology (SOBOPE) promotes and stimulates actions in order to improve the diagnostic possibility, the treatment, as well as the follow-up of the child with cancer. The dissemination of diagnostic and therapeutic guidelines through protocols carried out by Brazilian cooperative groups, which reflect our reality, generates better care for the child at a national level. In our country, the estimation is of 10,000 new cases/year of cancer in individuals aged less than 15 years, and we count on 174 cancer centers addressed to children and adolescents. In the state of São Paulo, 1,000 cases/year are estimated, with 20 specialized centers to treat this type of cancer. Many of these are also centers that train people, including medical and multiprofessional residency, therefore replicating qualified professionals to all corners of Brazil.

Besides, the medical-scientific advances in this field are remarkable. The improvement in the diagnosis with the immunohistochemical techniques, flow cytometry, molecular biology, cytogenetics and imaging is leading to increasing knowledge of these diseases, therefore contributing with the individualization of the treatment with new concepts, from the use of high doses of polychemotherapy until metronomic therapies, as well as complex surgeries and radiotherapy. This was possible thanks to support therapy, such as the modern antiemetics, nutritional, anti-infectious, respiratory, psychological, physical therapy efforts etc. The cure rates of specialized centers, such as the Institute of Pediatric Oncology of Grupo de Apoio ao Adolescente e à Criança com Câncer (GRAACC/IOP) surpass 70%.

We are following what we have learned from the experience of other countries: estabilishment of cooperative groups, investment in research, technology and, mainly, education.^3^ ^,^ ^4^

Classes of signs and symptoms for professors and health professionals, and the teaching of Oncopediatrics in courses addressed to health professionals, contribute with the early diagnosis.

However, the diagnosis depends on the clinical suspicion and on the periodical evaluation of the child, usually a responsibility of the pediatrician dedicated to primary health care. This professional is essential for the diagnosis and the proper referral for the child with cancer. Therefore, the publication of clinical manifestations of tumors in Pediatric journals is a good example of the collaboration in the scope of knowledge, essential pillar to improve pediatric oncology in Brazil.
